# Influence of TPU/EVA Phase Morphology Evolution on Supercritical Carbon Dioxide Extrusion Foaming

**DOI:** 10.3390/polym15143134

**Published:** 2023-07-24

**Authors:** Jun-Wei Du, Tian-Tian Zhou, Rong Zhang, Sheng-Fei Hu

**Affiliations:** Hubei Provincial Key Laboratory of Green Materials for Light Industry, Hubei University of Technology, Wuhan 430068, China; 502100002@hbut.edu.cn (J.-W.D.); 502100001@hbut.edu.cn (T.-T.Z.); zhangrong@hbut.edu.cn (R.Z.)

**Keywords:** thermoplastic polyurethane, phase morphology, melt strength, cell density

## Abstract

Ethylene-vinyl acetate copolymer (EVA) was added at different contents to the thermoplastic polyurethane (TPU) matrix to form a non-compatible blending system, and foaming materials with high pore density were prepared using the supercritical carbon dioxide extrusion method. The influence of the phase morphology and crystal morphology of the TPU/EVA blend on its foaming behavior was studied. The results show that EVA changed the phase morphology and crystal morphology of the blends, leading to the improved melt viscosity and crystallinity of the blend system. At the same time, interfacial nucleation increases the density of cells and decreases the cell thickness and size, which is beneficial for improving the foaming properties of the blends. For the EVA content of 10% (mass fraction), the cell size is small (105.29 μm) and the cell density is the highest (3.74 × 10^6^ cells/cm^3^). Based on the TPU/EVA phase morphology and crystal morphology, it is found that the sea-island structure of the blend has better foaming properties than the bicontinuous structure.

## 1. Introduction

Compared to polypropylene (PP), polyethylene (PE) and other foam materials, thermoplastic polyurethane (TPU) foam has good tensile strength, abrasion resistance and buffering performance, leading to its wide use in automobiles, packaging, construction, electronic equipment and other fields [1,2]. With the increasing maturation of the supercritical carbon dioxide (scCO_2_) foaming technology, the use of supercritical carbon dioxide foaming technology for the preparation of TPU foam has been extensively studied [3,4,5,6,7,8,9]. Under supercritical CO_2_, the crystallinity of TPU and the melt strength is low, making the cells prone to rupture, collapse and merge. The blending method can improve the rheological properties and thermal properties of TPU, thereby improving the cell morphology of the foaming material, as has been shown in several recent studies. Nalawade et al. used high-pressure infrared spectroscopy to study the effect of CO_2_ on the crystallization properties of TPU and found that CO_2_ can induce the crystallization of the hard segment of TPU and enhance the melt strength of TPU [10]. To further improve the foaming properties. Huang et al. added polytetrafluoroethylene (PTFE) for blending and found that PTFE forms a fiber structure in the TPU matrix that acts as a physical cross-linking point to enhance the strain hardening behavior and the melt strength of the TPU material, leading to a significant improvement in the TPU foam performance [11]. Zhang et al. used the reactive blending of TPU/acrylonitrile butadiene styrene (ABS) under the action of maleic anhydride (MAH) and dicumyl peroxide (DCP) to adjust the melt viscosity and melt strength of the blend and improve the cell shape and shrinkage resistance of the blend [12]. However, the TPU foam material also has a low cell density, resulting in a small expansion ratio.

Compared to TPU, ethylene-vinyl acetate copolymer (EVA) has higher solubility for CO_2_ and good foaming performance [13,14,15]. Jacobs et al. studied the effects of temperature and pressure on EVA supercritical CO_2_ foaming and found that when the temperature was close to the melting point, the cell density was increased, and a foam material with a small cell size and uniform distribution was obtained [16]. Maiti et al. studied the influence of the phase morphology of the EVA/polybutadiene rubber (BR) blend on the foaming performance and found that the phase morphology affects the open and closed porosity of the cells; they also prepared a high-open-porosity EVA foam material [17]. Rodriguez-Perez et al. studied the foaming properties of EVA/starch blends and found that starch acts as a bubble nucleation center to increase the cell density of the foamed material, leading to an improvement in the mechanical properties of EVA [18].

However, TPU and EVA blended and foamed by supercritical CO_2_ extrusion have been rarely reported. Such blending may be a good strategy for obtaining foams with improved performance and can build on the recent progress in the blending of non-foaming TPU and EVA. Dutta et al. studied the influence of the two-phase morphology of the TPU/EVA blends on the mechanical properties and found that the droplet morphology has a greater interfacial energy than the co-continuous structure. The stress can be effectively transferred between the matrix and the dispersed phase, endowing the blend with good mechanical properties [19]. Ma et al. studied the influence of EVA dispersibility on the mechanical properties of TPU/EVA and found that the EVA phase size is small and its dispersibility is good, improving the compatibility of the TPU/EVA blend, which in turn improves the mechanical properties of the blends [20].

The goal of the present work is to prepare a TPU foaming material with a high foaming density. To achieve this goal, EVA is added to the TPU matrix. The structure of the EVA dispersed phase and the TPU matrix phase formed in the blend system can promote the nucleation of the foaming pores, so as to achieve excellent foaming characteristics. The effects of the phase morphology and crystal structure on the rheological characteristics, crystallization characteristics and foaming behavior of TPU/EVA blends are studied by changing the blending ratio to form different phase morphology and crystal morphology. The obtained results identify the conditions for the preparation of a TPU foam material with a high cell density through a simple blending method.

## 2. Experimental

### 2.1. Materials

TPU (9385A) and EVA (260) were purchased from Desmopan Germany (Taibei, Taiwan, China) and DuPont (Wilmington, Delaware, USA), respectively. The melt flow rates (MFR) of TPU and EVA were 20 g/10 min (200 °C, 10 kg) and 6 g/10 min (190 °C, 2.16 kg), respectively. Carbon dioxide (CO_2_) with a purity of 99.5% supplied by the Wuhan Gas company (Wuhan, China) was used as the blowing agent.

### 2.2. Sample Preparation

**Figure 1:**
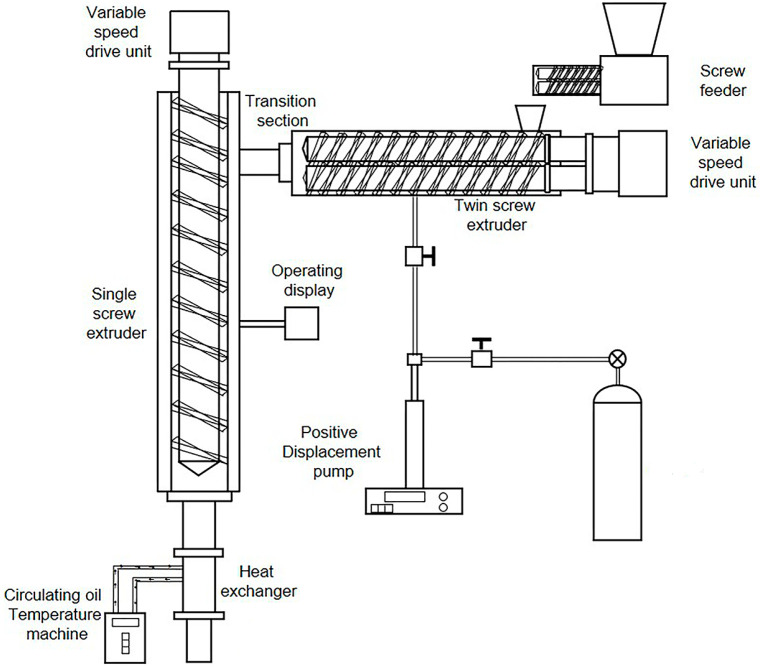
Schematic diagram of the serial extruder foaming system.

Prior to blending, TPU and EVA were dried at 40 °C for 12 h. The dried TPU and EVA were mixed in different proportions (Table 1). A foaming machine (Hubei University of Technology, Wuhan, China) was used to prepare the TPU/EVA foam materials (Figure 1). The temperatures of the twin-screw extruder and the single-screw extruder were set to 220 and 170 °C, respectively, and the pressure of the CO_2_ intake section was 8 MPa. The extruded TPU/EVA foaming samples were collected, cleaned with deionized water, and then dried in a vacuum drying oven at 45 °C for 12 h.

### 2.3. Characterization

#### 2.3.1. Morphological Study

The surface morphology of the blends was examined using a JOEL JSM 6390LV Digital Scanning Electron Microscope (SEM, JEOL, Tokyo, Japan) with the acceleration potential of 3 kV. All of the blends were cryofractured in liquid nitrogen to avoid any possibility of phase deformation during the cracking process. For each sample, the cryofractured surface was etched in chloroform for 1.5 h order to remove the EVA phase of the blends. After adequate drying for 3 h at room temperature, the etched surface was gold sputtered and then observed by SEM.

#### 2.3.2. Rheological Properties

A stress-controlled rotational rheometer (AR2000EX, TA, New Castle, DE, USA) was used to study the rheological properties of the TPU/EVA blends at 170 °C (diameter 25 mm, gap 1 mm). The angular velocity was set to 1.0–100 rad/s and the strain was 1% (selected for the strain sweep test of the rheological sample at a fixed frequency of 6.28 rad/s).

#### 2.3.3. Differential Scanning Calorimetry (DSC) 

A differential scanning calorimeter (American PE company DSC8500, Waltham, MA, USA) was used to study the melting crystallization behavior of the TPU/EVA blends with different EVA contents. In nitrogen atmosphere, the samples were heated from 0 to 200 °C at a heating rate of 20 °C/min, kept for 5 min to eliminate the thermal history, and then cooled to 0 °C at a cooling rate of 20 °C/min, kept for 2 min, and finally reheated to 200 °C at a heating rate of 20 °C/min. The mass of each sample was approximately 3 mg.

#### 2.3.4. X-ray Diffraction (XRD) 

X-ray diffraction (XRD, Bruker D8 ADVANCE, Bruker, Karlsruhe, Germany) was used to study the crystal structure of the TPU/EVA blends at different temperatures. The scanning temperature range was 40–210 °C, the scanning angle 2*θ* was 5–90°, and the scanning speed was 5°/min.

#### 2.3.5. Polarizing Microscope (POM) 

A polarizing microscope (POM, Leica DM 4500P, Leica, Wetzlar, Germany) was used to study the crystal morphology of the TPU/EVA blends with different EVA contents. A sample was rapidly removed immediately after the extruded foam comes out of the die and was pressed into a tablet to observe the crystal shape.

#### 2.3.6. Foaming Characteristics

In this study, the foam morphology was investigated using a scanning electron microscope (SEM, QUANTA Q400-FEI, Thermo Fisher Scientific, Waltham, MA, USA). To effectively preserve the morphology of the foam samples, a sudden cooling brittle fracture technique employing liquid nitrogen was implemented. Subsequently, a thin layer of gold was applied to the foam sections as a coating. Following these preparations, the vesicular architecture of the sections was thoroughly investigated using a scanning electron microscope. 

The quantification of bubble size was performed by employing Image pro-plus software to determine the number average bubble diameter. The number average vesicle diameter (*d*) and vesicle density (*Nc*) were subsequently calculated using the following equations:

(1) $$d=\sum d_{i}n_{i}/\sum n_{i}$$

(2) $$N_{c}=6\times 10^{12}\left(\rho /\rho_{f}-1\right)/\pi d^{3}$$

where *d* is the number average bubble diameter in µm; $n_{i}$ is the number of bubbles with an equivalent diameter of $d_{i}$; $N_{c}$ is the density of bubbles in cells/cm^3^; $\rho_{f}$ and ρ are the density of foamed and unfoamed samples, respectively (measured by drainage), g/cm^3^.

## 3. Results and Discussion

### 3.1. Morphology

**Figure 2:**
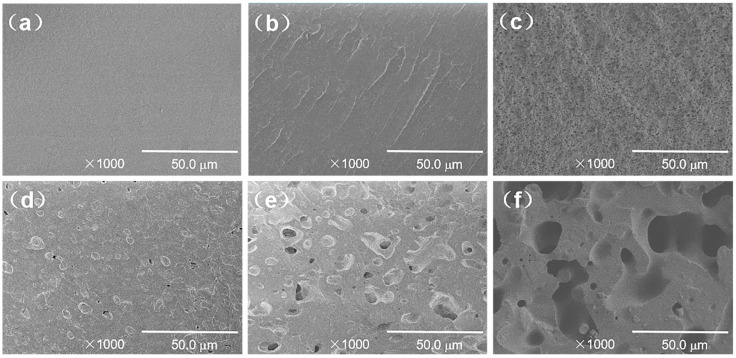
SEM micrographs of the fracture surface of the TPU/EVA blends with different EVA contents: (**a**) TPU, (**b**) TE-5, (**c**) TE-10, (**d**) TE-12, (**e**) TE-13 and (**f**) TE-15.

**Figure 3:**
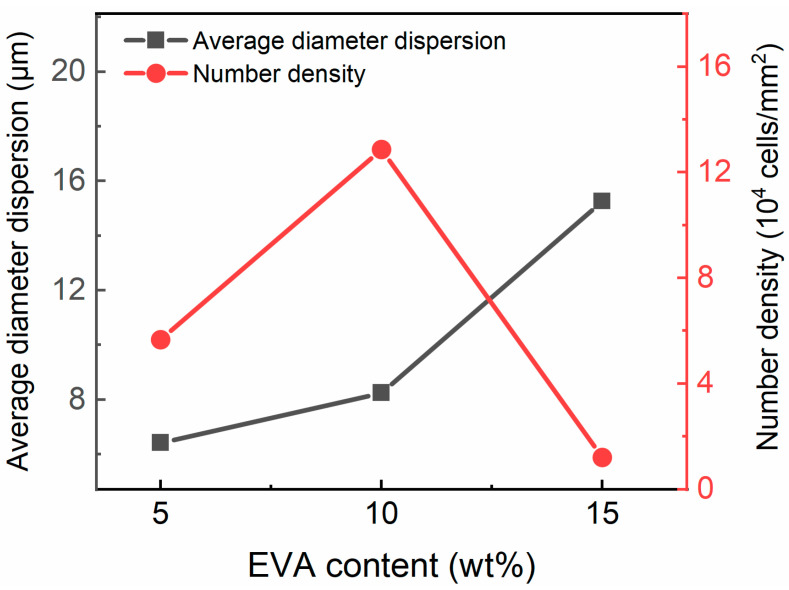
Average diameter and density of the dispersed phase in the TPU/EVA blends with different EVA contents.

Figure 2 shows an SEM image of the TPU/EVA blends etched with chloroform (CHCl_3_). It is observed from Figure 2 that the pure TPU region is smooth and flat, the TPU/EVA blend presents a typical sea-island two-phase morphology, and EVA is uniformly dispersed in the TPU matrix as spherical particles. When the EVA content reaches 12% (Figure 2d), a short continuous phase structure appears in the EVA dispersed phase, and the phase morphology of the blend begins to change to a bicontinuous phase structure. With a further increase in the EVA content, the EVA presents a clear dual continuous phase structure (Figure 2f) with a clear interface between the two phases, indicating that TPU and EVA are incompatible blending systems. The average particle size and density of the dispersed phase particles calculated using the Image-J software are plotted versus the EVA content in Figure 3. It is observed that the average particle size of the EVA dispersed phase shows an increasing trend, while the number of the particles of the dispersed phase first increases and then decreases. When the EVA content is 10%, the density of the dispersed phase particles reaches the maximum value of 1.287 × 10^5^ particles/mm^2^, and the average particle size of the EVA dispersed phase is only 2.738 μm at this time, indicating that the blend contains a large number of interfaces. Since the energy required for cell nucleation at the interface of the two phases is small, this promotes cell nucleation, and the size and density of the dispersed phase affect the cell morphology [21]. When the EVA content is further increased, the phase morphology of the TPU/EVA blend changes from a two-phase sea-island structure to a bicontinuous morphology, the average particle size of the dispersed phase becomes larger, and the number of dispersed phase particles decreases. This is due to the agglomeration of the EVA particles that leads to the larger size of the dispersed phase particles and a decrease in the number of particles [22].

### 3.2. Rheological Properties of TPU/EVA Blends

The foaming performance characteristics of the materials are directly affected by their rheological properties. During the foaming process, the viscoelastic behavior of polymers strongly affects the cell morphology [23]. When the cell grows, the polymer melt will be subjected to a complex deformation that requires sufficient deformation space. Without such space, the cell cannot grow, the expansion ratio is limited, and the polymer melt must have a sufficiently high melt strength to ensure that the cells will not rupture or collapse.

**Figure 4:**
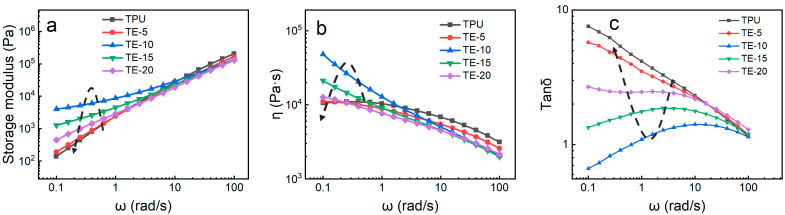
Rheological curves of TPU/EVA blends with different EVA contents at 170 °C: (**a**) storage modulus, (**b**) complex viscosity and (**c**) tan (δ).

Figure 4 shows the curve of the rheological properties of the TPU/EVA blend. It is observed from the storage modulus (G’) curve (Figure 4a) that the G’ of the blend material increases with increasing frequency (ω). In the low-frequency region, the G’ of the blend first increases and then decreases with increasing EVA content, and the G’ of TE-10 reaches the maximum value. For incompatible TPU/EVA blends, the increase in G’ in the low-frequency region is attributed to the relaxation effect of the blend system [24]. As the EVA content further increases, the relaxation time of the blended system decreases, and the EVA dispersed phase does not have sufficient energy to recover the relaxation process. The energy generated by the deformation is mainly consumed in the TPU matrix. Therefore, the G’ of the TPU/EVA blend system decreases with increasing EVA content after TE-15 [25]. It is observed from the complex viscosity (*η*) curve (Figure 4b) that as the frequency increases, the *η* of the blend decreases, showing a phenomenon of shear thinning. With increasing EVA content, the *η* of the blend first increased and then decreased, and a pronounced change in the *η* of the blend in the low-frequency region was observed, with η reaching the maximum value for the TE-10 blend. With increasing EVA content, the value of *η* initially increases because EVA is uniformly dispersed in the TPU matrix and their molecular chains are strongly entangled, giving rise to strong interactions between EVA and TPU [26]. With the further increase in the EVA content, the *η* of the blended material decreases as the size of the EVA phase particles increases, and the interface shows larger slip. In low-frequency shearing, TE-10 shows the best elastic behavior and the longest relaxation time [27]. The uniformly dispersed EVA plays an important role in increasing *η* and G’. It is observed from the curve of tan δ (Figure 4c) that, compared to pure TPU, the tan δ of the blend material is reduced and its foamability is improved, so that the smallest tan δ is obtained for the TE-10 material.

### 3.3. Crystallization Properties of TPU/EVA Blends

**Figure 5:**
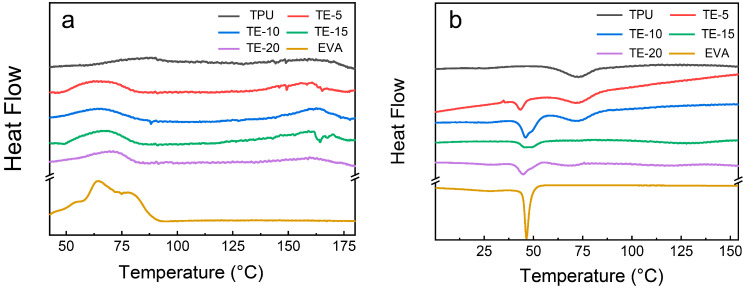
DSC curves of the TPU/EVA blends with different EVA contents: (**a**) melting curve and (**b**) cooling curve.

Figure 5a,b show the DSC melting curve and crystallization curves of the TPU/EVA samples with different EVA contents, respectively. The parameters extracted from the DSC results for the TPU/EVA samples are listed in Table 2. Using these data, the trend of the crystallization characteristics of the prepared TPU/EVA foams can be analyzed and compared.

Figure 5a shows that the melting curve of the pure TPU material has two melting peaks at approximately 75 and 160 °C, respectively. The peak at 75 °C is the melting peak of the hard segment crystal region of TPU, and the peak at 160 °C corresponds to the complete melting of TPU [28]. The melting curve of the pure EVA material shows a single melting peak that appears at approximately 63.25 °C. With the increase in the EVA content, the melting peak of TPU at 75 °C shifts to the left, and the melting temperature range becomes wider. The theoretical crystallinity of each material can be calculated following the method used in previous work [29]. An examination of the calculation values presented in Table 2 shows that the melting peak, melting enthalpy and crystallinity of the TPU/EVA blend first increase and then decrease, with the highest crystallinity obtained for the EVA content of 10%.

As shown in Figure 5b, the crystallization curves of pure TPU and EVA show only a single crystallization peak, while the TPU/EVA blend material exhibits two crystallization peaks at approximately 75 and 49.8 °C, respectively. With the increase in the EVA content, the intensity of the crystallization peak at 49.8 °C first increased and then decreased, with the highest intensity reached for the TE-10 sample. For incompatible blending systems, crystal formation can proceed through homogeneous nucleation and heterogeneous nucleation [30,31]. Since TPU has low crystallinity, the crystal formation in the TPU/EVA blend system is mainly driven by heterogeneous nucleation. Due to the lower energy required for crystal nucleation at the two-phase interface, the heterogeneous nucleation of the crystals can be initiated, and the size and density of the dispersed phases will affect the degree of crystallization of the blends [32,33,34]. The highest crystallinity observed for the TE-10 sample is due to the increase in the average particle size of the EVA dispersed phase, and the increase in the density of the dispersed phase. This improves the area of the interfacial layer in the blend and enhances its crystallinity. When the EVA content increases further, the average particle size of the dispersed EVA phase increases, while the density of the dispersed phase particles decreases, corresponding to a sharp decrease in the number of interfacial layers of the blend that gives rise to a sharp decrease in the number of heterogeneous nucleation sites in the blends. This leads to the decreased crystallinity of the blend system. In the process of bubble growth and molding, the crystallization behavior of the blend material is improved, endowing the blend material with higher melt strength, and reducing bubble pore merging, rupture and collapse. This promotes the formation of bubble pores. Thus, the introduction of EVA changed the crystallization properties of the blend materials and affected their foaming properties.

### 3.4. XRD Analysis of TPU/EVA Blend

**Figure 6:**
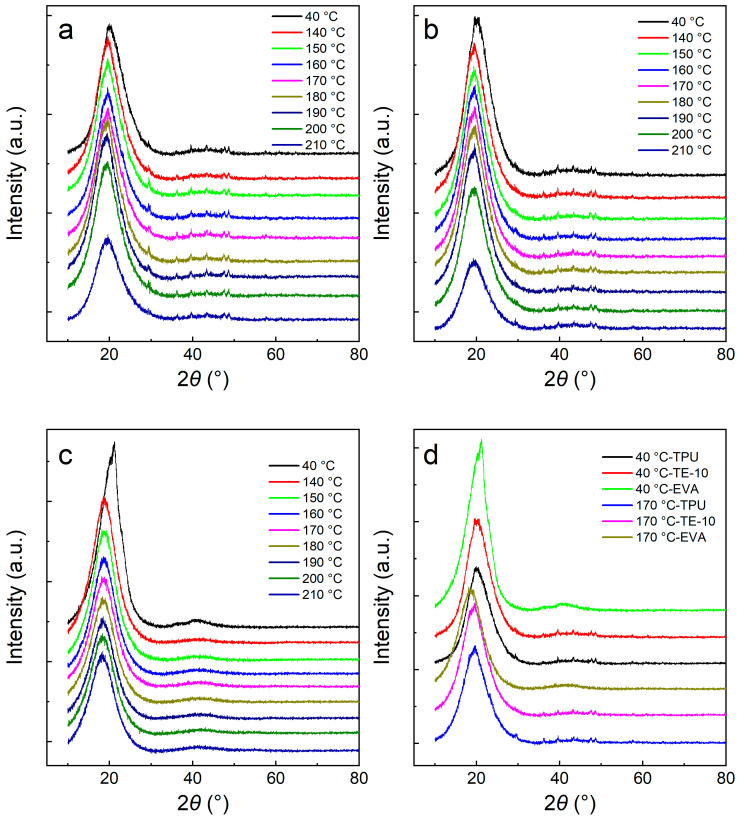
XRD patterns of the TPU/EVA blends at different temperatures: (**a**) TPU, (**b**) TE-10, (**c**) EVA and (**d**) XRD patterns of the TPU/EVA blends at different EVA contents.

The crystalline region of TPU is mainly formed by hard segments, and the amorphous region is formed by soft segments. Due to the different lengths of the hard segments, crystal particles show a strong variation in the crystal particle size. The crystals formed by the short hard segment melt at a low temperature, and the crystals formed by the long hard segment melt at a high temperature [35]. Figure 6 shows the XRD patterns of the TPU/EVA blends at different temperatures. It is observed from Figure 6a,b that the characteristic diffraction peak of TPU at 2*θ* = 19.6° is also observed for TE-10, indicating that the addition of EVA does not change the crystal form of TPU. As the temperature increases, the characteristic diffraction peak intensity of the blend decreases slightly due to the melting of the crystals of the short hard segment. As the temperature is raised to 210 °C, the characteristic diffraction peak intensity of the blend decreases significantly due to the melting of the TPU/EVA blend crystals and the peak. At 210 °C, the crystals are melted completely and the characteristic diffraction peaks of TPU and TE-10 are amorphous peaks [36]. These results show that at the foaming temperature of 170 °C, crystals are still present in the TPU/EVA blend, but the number of crystals varies with the temperature. As observed from Figure 6c, when the temperature is 40 °C, the characteristic diffraction peak of EVA appears at 2*θ* = 21.2°, while for the temperature of 140 °C, the peak is found at 18.5°, indicating that the crystal form of EVA has changed. Additionally, the peak intensity of EVA is significantly weakened at 140 °C relative to 40 °C. When the temperature is further increased, the characteristic diffraction peaks of EVA did not change, indicating that the EVA crystals had completely melted at 140 °C. It is observed from Figure 6d that at 170 °C, the diffraction peak intensity of TE-10 is stronger than that of TPU, indicating that the crystallinity of TE-10 is higher than that of TPU. This shows that the addition of EVA improves the crystallinity of the blend and enhances the crystallinity of TPU. When the foaming temperature is 170 °C, the crystallinity of the blend is increased, the melt strength of the blend is improved, and the phenomenon of cell cracking and collapse of the blend is reduced.

### 3.5. Crystal Morphology of TPU/EVA Blend

**Figure 7:**
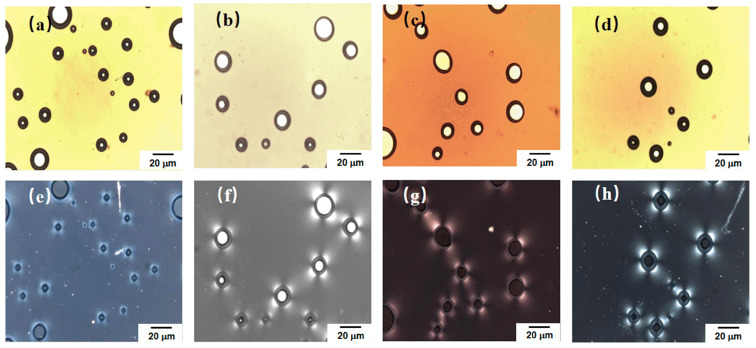
Crystal morphology for different EVA contents, observed with a polarizer: (**a**) TPU, (**b**) TE-5, (**c**) TE-10 and (**d**) TE-15. Under non-polarized light: (**e**) TPU, (**f**) TE-5, (**g**) TE-10 and (**h**) TE-15.

Figure 7 shows polarized optical microscopy images of the extruded foam samples of the TPU/EVA blend. Figure 7a–d show the morphology of the foamed sample under non-polarized light after compression. It is observed that there are still a few cells in the sample, and the cells are either spherical or ellipsoidal. An examination of the images obtained with the polarizer and presented in Figure 7e–h shows that the TPU/EVA blend system is centered on the cell, and an obvious Maltese cross-phenomenon is present, but there is no Maltese cross-phenomenon in the regions without bubbles. This indicates that bubbles play a key role in either homogenous nucleation or interfacial nucleation in the composite system, forming spherulites centered on the bubble [37,38,39]. At the same time, upon changing the EVA content, the TPU/EVA blend system can form obvious spherulites, and the degree of crystallinity is higher than that of pure TPU. This shows that the addition of EVA and supercritical CO_2_ are beneficial for promoting the crystallization performance of the TPU/EVA blend system.

**Figure 8:**
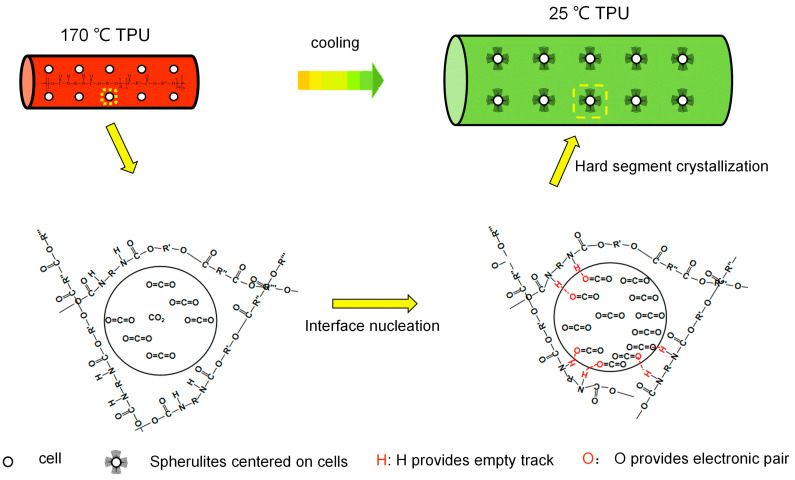
Interface nucleation process for the formation of spherulites.

This may be due to the Lewis acid–base interaction between the CO_2_ in the bubble and the imino (N-H) in the TPU hard segment during the bubble growth. In these functional groups, the O atom in CO_2_ provides an electron lone pair, and the H atom in N-H provides an unoccupied orbital that can interact with each other [40,41,42]. Due to the Lewis acid–base interaction between the H and O atoms, the TPU hard segment is attached to the bubble interface, resulting in interface nucleation (as shown in Figure 8). When the temperature decreases, the hard segments begin to crystallize around the bubbles, grow outward, and finally form spherulites centered on the bubbles. The formation of spherulites endows the cell walls with higher strength, reduces the size of the cells, inhibits the escape of the gas, reduces cell rupture, collapse and other phenomena, stabilizes the cell structure, and improves the cell morphology.

### 3.6. Crystallization Kinetics of TPU/EVA Blend

The crystallization process of the TPU/EVA blends is closely related to cell growth and solidification. Both the nucleation method and growth type of crystals are beneficial for improving the melt strength of the polymer, endow the cell walls with higher strength, and promote the solidification of the cells. The Avrami equation can describe the crystallization process of the polymer crystals [34,43], where the relative crystallinity ($X_{t}$) is a function of time *t* given by Equations (3) and (4):

(3) $$X_{t}=\frac{x_{t}}{x_{\infty }}=\frac{\int_{0}^{t}\left(d\Delta H/dt\right)dt}{\int_{0}^{\infty }\left(d\Delta H/dt\right)dt}=\frac{A_{t}}{A_{\infty }}$$

where *t* is the crystallization time, $x_{t}$ and $x_{\infty }$ are the fractions of the amorphous state transformed into the crystalline state when the crystallization time is *t* and infinity, respectively, and $A_{t}$ and $A_{\infty }$ are the areas contained in the DSC curves during the time intervals of 0–*t* and 0–∞, respectively.

(4) $$ln\left[-ln\left(1-X_{t}\right)\right]=lnZ_{t}+nlnt$$

where $Z_{t}$ is the crystallization kinetic rate constant, and *n* is the Avrami constant.

The crystallization rate of the polymer can be reflected by the half-crystallization time ($t_{1/2}$, $X_{t}$). The crystallization of TPU/EVA blends is a non-isothermal crystallization process. It can be described by the Jeziorny normal equation [44], as shown in Equations (5) and (6):

(5) $$t_{1/2}={\left(ln2/Z_{t}\right)}^{1/n}$$

(6) $$lnZ_{c}=lnZ_{t}/\varphi$$

Using the DSC test data, the values of *n* and $Z_{c}$ of the corresponding component are calculated using Equations (5) and (6) and are shown in Table 3.

An examination of the data presented in Table 3 shows that the $t_{1/2}$ of the TPU/EVA blend system first decreases and then increases, with the smallest value obtained for the TE-10 sample. The initial $t_{1/2}$ decreases indicate that the addition of EVA content accelerates the crystallization rate of the blend and promotes the crystallization of the TPU/EVA blend. Then, when the EVA content is further increased, the $t_{1/2}$ of the blend gradually increases because the particles of the dispersed phase EVA become larger, reducing the relative surface area of the interface and the crystallization rate of TPU/EVA.

The Avrami constant n is closely related to the nucleation mechanism and growth mode of the blend crystals [43]. As shown in Table 3, the *n* value of pure TPU is between 1 and 2. For TPU, homogeneous nucleation is the key process and the crystal nucleus consists of macromolecular chains, so that the crystals mainly grow along a one-dimensional sheet pattern. With the addition of EVA, the change to *n* ≈ 3 for the blended material indicates that the crystal grows three-dimensionally in spherical manner, obtaining spherulites [44]. The addition of EVA and CO_2_ generates interfaces between EVA and TPU, and between CO_2_ gas and TPU. These interfaces lead to interface nucleation by adsorbing the polymer chains in the TPU melt, and arranging them in an orderly manner to form crystal nuclei that grow into spherulites. The *n* > 4 obtained for TE-10 indicates that in this case the crystals of the TPU/EVA blend mainly grow along the three-dimensional spherical pattern to form spherulites, and homogeneous nucleation also contributes [45]. The increased degree of crystallinity and the formation of spherulites increases the melt strength of the blend, supports the growth of cells, inhibits cell rupture and merging, and improves cell morphology.

### 3.7. Foaming Performance

During extrusion from the die, a sudden drop in the pressure destroys the equilibrium of the TPU/CO_2_ homogeneous system and forms bubble cores. Since the interfaces between TPU and EVA act as the cell nucleation centers, the free energy required for cell heterogeneous nucleation is lower than that of homogeneous nucleation. The bubbles are mainly formed by heterogeneous nucleation. The free energy required for heterogeneous nucleation ($\Delta G_{het}$) can be expressed by the Suh and Colton equations [21].

(7) $$\Delta G_{het}=\left(16\pi \sigma /\left(3\Delta P^{2}\right)\right)f\left(\theta \right)$$

(8) $$f\left(\theta \right)=\left(1/4\right)\left(2+cos\theta \right){\left(1+cos\theta \right)}^{2}$$

where ∆*P* is the pressure difference between the inside and outside of the bubble, *θ* is the wetting angle between the two-phase interface, 0 < θ < 1, 0 < f(θ) < 1. Calculations using Equations (7) and (8) show that there is a low-energy point between TPU, EVA and the bubble interface during the cell nucleation process that acts as the site for the initial nucleation of the bubbles [46].

**Figure 9:**
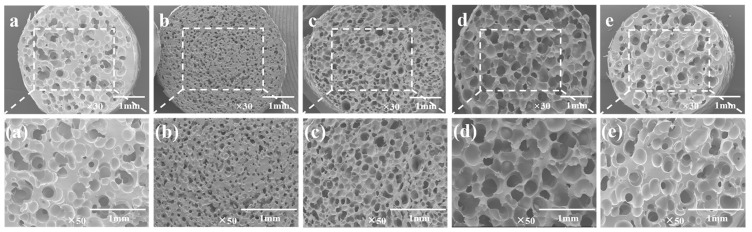
SEM images of the TPU/EVA blend foam materials with different EVA contents at 170 °C: (**a**) TPU, (**b**) TE-5, (**c**) TE-10, (**d**) TE-15 and (**e**) TE-20.

**Figure 10:**
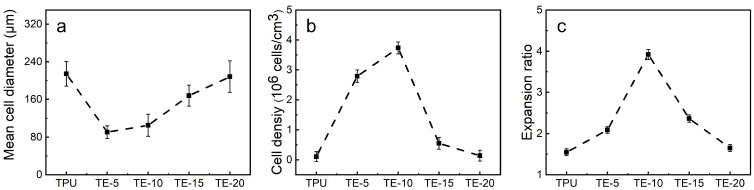
(**a**) Mean cell diameter, (**b**) cell density and (**c**) expansion ratio of TPU/EVA blends with different EVA content.

Bubble nucleation is the key step in the foaming process. The number of the nucleated bubbles determines the cell density of the foam material and indirectly affects the expansion ratio. In addition, the melt viscosity of the polymer and the solubility of CO_2_ will also affect the bubble morphology. Figure 9 shows SEM images of the TPU/EVA blend foam materials with different EVA contents at 170 °C. It is observed from the cell structure that the pure TPU foam sample exhibits cell rupture and merger, and the cell wall is thick. This is due to the low TPU melt viscosity and strength at 170 °C. During cell growth, the cell wall does not have sufficient strength and it is difficult to maintain the shape of the cell. The thickness of the cell wall of the foamed sample of the TPU/EVA blend is reduced and the number of cells is large. After the addition of EVA, the cell rupture and merger phenomenon of the TPU/EVA foam material is reduced. This is because the crystallization behavior of the TPU/EVA blend is enhanced to form spherulites centered on the cells, improving the blend. The melt viscosity and strength of the melt reduce the occurrence of cell merger and collapse, and improve the cell shape. The average cell diameter and cell density of the foamed samples of the TPU/EVA blend can be obtained from the images presented in Figure 9 and are shown in Figure 10. It is observed from the cell data that the average cell diameter first decreases and then increases with the increase in the EVA content, while the cell density shows the opposite trend. For the EVA content of 10% (mass fraction), the cell size is small (105.29 μm) and the cell density is the highest (3.74 × 10^6^ cells/cm^3^). For the TPU/EVA blend system, the cells are mainly nucleated at the interface, and the interface between TPU and EVA affects the number of cells. The phase morphology study shows that the highest number of dispersed EVA particles is obtained for the EVA content of 10%. Thus, the highest interface area and the highest likelihood of nucleation at the interface are obtained for this EVA content to maximize the cell density. When the EVA content is further increased, the TPU/EVA blend system changes from a sea-island two-phase structure to a bicontinuous phase structure. The EVA particles aggregate, the average particle size of the dispersed phase increases, and the number of dispersed phases decreases, reducing the relative area of the interface. Thus, the number of nucleation sites at the bubble interface decreases, and the cell density decreases as well. For 10% EVA content, the melt viscosity and crystallinity of the blend system are enhanced, thereby enhancing the melt strength of the blend. During the cell growth, the cell wall has higher strength, reducing the occurrence of cell rupture and merging. At the same time, a large number of interfaces are present, increasing the number of nucleation sites at the bubble interface and the cell density.

## 4. Conclusions

Ethylene-vinyl acetate copolymer (EVA) was added to the thermoplastic polyurethane (TPU) matrix to form an incompatible blend system. The TPU/EVA foam material prepared by supercritical carbon dioxide extrusion foaming has good cell size and cell density. Upon the addition of EVA to the TPU matrix, the phase morphology of the blend system changes from a sea-island two-phase structure to a bicontinuous phase structure. The melt viscosity and crystallinity of the blend system first increase and then decrease. Because the interface initiates the heterogeneous nucleation of cells, the cell density increases and the cell size decreases. At the same time, the crystals form spherulites centered on the cells, improving the melt strength of the blend and avoiding cell cracking. By changing the blending ratio, the number of cells and cell size can be controlled. For the EVA content of 10%, a foamed material with a cell size of 105.29 μm and a cell density of 3.74 × 10^6^ cells/cm^3^ is obtained. Due to the TPU/EVA phase morphology structure, the sea-island structure of the blend has better foaming properties than the bicontinuous structure. These research results provide guidance for the preparation of TPU foam materials with high cell density.

## Figures and Tables

**Table 1:** Compositions of the samples.

Sample	TPU (wt%)	EVA (wt%)
TPU	100	0
TE-5	95	5
TE-10	90	10
TE-12	88	12
TE-13	87	13
TE-15	85	15
TE-20	80	20
EVA	0	100

**Table 2:** Melting of TPU/EVA blends with different EVA contents.

Sample Designation	Melting Peak T_m_ (°C)	Melting Enthalpy ∆H (J/g)	Crystallinity (%)
TPU	159.3	2.46	1.6
TE-5	160.7	3.99	2.7
TE-10	162.6	8.57	5.8
TE-15	159.6	6.14	4.1
TE-20	159.1	2.77	1.8

**Table 3:** Non-isothermal crystallization kinetic parameters of the TPU/EVA blends with different EVA contents.

Sample	*n*	$t_{1/2}/\min$	$Z_{t}$	$Z_{c}$
TPU	1.52	2.68	0.16	0.83
TE-5	3.72	1.83	0.07	0.77
TE-10	4.20	1.67	0.08	0.78
TE-15	2.56	1.70	0.18	0.84
TE-20	2.61	1.72	0.17	0.84

## Data Availability

The raw/processed data required to reproduce these findings cannot be shared at this time due to technical or time limitations.
